# A Filtering Method to Generate High Quality Short Reads Using Illumina Paired-End Technology

**DOI:** 10.1371/journal.pone.0066643

**Published:** 2013-06-17

**Authors:** A. Murat Eren, Joseph H. Vineis, Hilary G. Morrison, Mitchell L. Sogin

**Affiliations:** Josephine Bay Paul Center for Comparative Molecular Biology and Evolution, Marine Biological Laboratory, Woods Hole, Massachusetts, United States of America; Georgia Institute of Technology, United States of America

## Abstract

Consensus between independent reads improves the accuracy of genome and transcriptome analyses, however lack of consensus between very similar sequences in metagenomic studies can and often does represent natural variation of biological significance. The common use of machine-assigned quality scores on next generation platforms does not necessarily correlate with accuracy. Here, we describe using the overlap of paired-end, short sequence reads to identify error-prone reads in marker gene analyses and their contribution to spurious OTUs following clustering analysis using QIIME. Our approach can also reduce error in shotgun sequencing data generated from libraries with small, tightly constrained insert sizes. The open-source implementation of this algorithm in Python programming language with user instructions can be obtained from https://github.com/meren/illumina-utils.

## Introduction

Massively parallel sequencing (MPS) of 16S rRNA gene amplicons has revolutionized our understanding of microbial ecology and diversity. Initial marker gene studies that collected several thousand amplicon sequences per sample using the Roche GS20 platform revealed that microbial diversity significantly exceeded estimates based upon traditional taxonomy and capillary electrophoresis (CE) sequencing [1], [2]. Low-abundance taxa that MPS marker genes detect through the collection of large molecular datasets relative to CE sequencing studies account for most of the expanded diversity. Yet, random sequencing errors can inflate the number of observed Operational Taxonomic Units (OTUs) in analyses that rely upon *de novo* clustering methods and therefore challenge the accuracy of microbial diversity estimates [3], [4].

Relative to pyrosequencing on the Roche GS20 and FLX sequencers, the Illumina platform offers significantly increased sequencing depth and a robust paired-end sequencing technology that recovers DNA sequence from the both ends of a single DNA template. Quality filtering methods for Illumina reads generally rely upon machine-reported Q-scores and empirically defined thresholds to eliminate noise. Varying the stringency of these thresholds changes the sensitivity and specificity of the outcome, without guaranteeing an accurate basecall [5], [6]. Agreement between overlaps of paired-end reads in MPS analyses can guide quality filtering to increase sequence accuracy. For marker gene analyses, the selection of primer sites within well-conserved regions will control the library insert size and specify the extent of overlap between paired-end reads. Alternatively, the insert size of shotgun genomic or metagenomic libraries can be constrained to a narrow range by size selection that also can control the extent of overlap for the forward and reverse reads.

Zhou *et al*. [7] previously described the use of overlapping reads to improve amplicon sequence quality, and Masella *et al*. [8] recently developed a fast aligner (PANDAseq) for overlapping paired-end reads that employs Q-scores to solve disagreements between mismatches and retain a larger number of high-quality reads compared to naïve approaches. However, to the best of our knowledge, popular Q-score based filtering methods for Illumina reads have not been benchmarked against methods that benefit from the overlapping regions of paired-end reads for quality filtering.

Here we present a set of multiplexing fusion primers, a library preparation method and associated analysis software to generate very high quality short reads on Illumina platforms using paired-end technology. We evaluate this strategy by applying it to the V6 region of the 16S rRNA gene amplified from control and environmental samples, and compare the quality control procedure we present with two other quality filtering approaches [5], [6] to discuss the sensitivity and specificity limitations of Q-score-based quality filtering methods.

## Materials and Methods

### Sampling and Library Preparation

**Figure 1:**
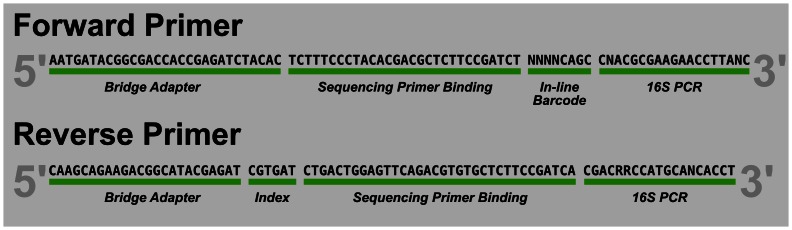
Structure of V6 fusion primers used to generate amplicon libraries for Illumina sequencing.

We amplified three separate replicates of the V6 region of ribosomal RNAs from *Escherichia coli* (*E. coli*) genomic DNA isolated from pure culture and from 10 metagenomic microbial DNA samples isolated from raw sewage. Custom fusion primers for PCR consisted of the Illumina adaptor, 12 different inline barcodes (forward primer) or 8 dedicated indices (reverse primer), and conserved regions of the V6 sequence (Figure 1). This use of 96 unique barcode-index combinations allows multiplexing 96 samples per lane. Paired indices with dual indexing reads could further increase the level of multiplexing. For each of the 33 libraries, we carried out the PCR in triplicate 33 uL reaction volumes with an amplification cocktail containing 1.0 U Platinum Taq Hi-Fidelity Polymerase (Life Technologies, Carlsbad CA), 1X Hi-Fidelity buffer, 200 uM dNTP PurePeak DNA polymerase mix (Pierce Nucleic Acid Technologies, Milwaukee, WI), 1.5 mM MgSO4 and 0.2 uM of each primer. We added approximately 10–25 ng template DNA to each PCR and ran a no-template control for each primer pair. Cycling conditions were: an initial 94°C, 3 minute denaturation step; 30 cycles of 94°C for 30s, 60°C for 60s, and 72°C for 90s; and a final 10 minute extension at 72°C. The triplicate PCR reactions were pooled after amplification and purified using a Qiaquick PCR 96-well PCR clean up plate (Qiagen, Valencia CA). Purified DNA was eluted in 30 uL of Qiagen buffer EB. PicoGreen quantitation (Life Technologies, Carlsbad CA) provided a basis for pooling equimolar amounts of product. After size-selecting products of 200–240 bp on 1% agarose using Pippin Prep (SageScience, Beverly MA), we employed qPCR (Kapa Biosystems, Woburn MA) to measure concentrations prior to sequencing on one lane of an Illumina Hiseq 100 cycle paired-end run. The remaining 90% of the lane was dedicated to PhiX DNA and served as the run control. The combination of CASAVA 1.8.2 to identify reads by index and a custom Python script that resolved barcodes demultiplexed the datasets.

### Complete Overlap Quality Filtering

Requiring 100% consensus between the overlapping regions of the forward and reverse paired-end sequencing reads eliminates the vast majority of sequencing errors. Both the first and second reads in paired-end sequencing runs on an Illumina platform using 100 cycles in each direction will span the entire V6 region and extend 15–20 nt into the proximal and the distal PCR primer sites. The quality filtering procedure takes advantage of the complete overlap of the forward and reverse reads to retain or discard the paired-end reads according to their 100% consensus between the primer sites. The required quality control operations for each paired-end sequencing read include: 1) compute the reverse-complement for the second read; 2) Within the last 30 nt of both the forward read and the reverse complemented second read search for the initial 6 nucleotides of the distal V6 primer; 3) Discard sequence pairs that do not perfectly match the initial 6 nt of distal primer in both reads; 4) Within the initial 40 nt of both the forward read and the reverse complemented second read search for the initial 10 nucleotides that matches a consensus sequence for the four proximal primers (967F-AQ, 967F-UC3, 967F-PP and 967F-UC12; see Table S1 for details); 5) If either of the reads fail to match the consensus, discard the pair; 6) Trim the proximal (including its barcode) and distal primers from each read; 7) Retain the sequence pair as a quality-passed V6 sequence if they share 100% consensus between the primer sites. The URL https://github.com/meren/illumina-utils provides access and user instructions for the open-source implementation of the V6 complete overlap analysis program that analyzes raw output files of CASAVA version 1.8.0 or higher. It also produces graphics for visualization of machine-reported quality scores for reads that survived the quality filtering and reads that failed, and reports overall statistics about the analysis for quality assurance.

### Quality Score Based Quality Filtering

To compare results of complete overlap quality filtering with quality-based filtering, we analyzed raw sequence data using two recently published methods that rely on Q-scores. We used the method described by Bokulich *et al.* [5] with suggested parameters *p* = 0.75, *q* = 3, *r* = 3, *and n* = 0, where *p* defines the ratio of the trimmed read length relative to the original read length, *q* is the Q-score threshold that classifies a base as low-quality, *r* is the minimum number of consecutive bases with Q-scores lower than *q* from the beginning of the read to identify the location of quality trimming, and *n* is the number of ambiguous bases permitted in a quality trimmed read. We also tested the quality filtering technique recommended by Minoche *et al.* [6] using a default length value p = 0.75 as recommended in [5]. We applied the quality-based filtering methods independently to each of the reads in a paired-end sequence. If either of the reads exhibited low quality by these criteria, we discarded both reads. For the convenience of the reader, we refer to these two methods as "Minoche" and "Bokulich" throughout the manuscript.

### Clustering

To compare OTU clustering performance of each quality filtering method, we clustered each sample with QIIME (v1.5) [9] using the default UCLUST method [10] and a 97% similarity threshold for OTU formation with minimum cluster size of 2. Because complete overlap analysis consistently yielded the smallest number of reads for each sample (Table S2), we randomly subsampled reads generated by Bokulich and Minoche 5 times to prevent any bias in clustering results and clustered the result of each subsampling independently to report the mean number of OTUs for each sample.

## Results

**Figure 2:**
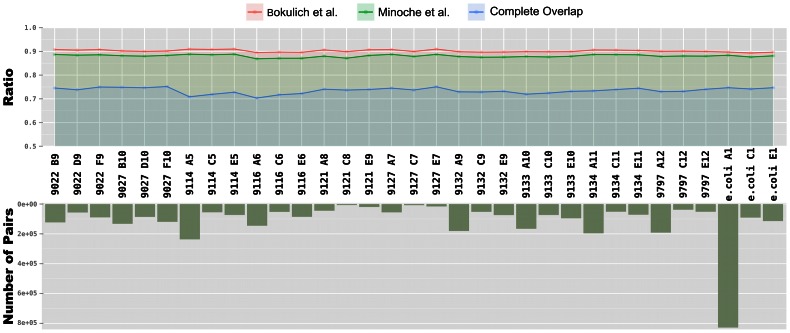
Comparison of three filtering methods. The top panel shows the ratio of pairs identified as low quality versus all pairs analyzed for each method. The total number of pairs in each dataset is shown in the bottom panel.

We used complete overlap analysis, which relies upon consensus between paired-end reads rather than Q-scores, to identify sequencing errors in an Illumina HiSeq dataset of V6 amplicon sequences from 33 samples. From a total of 3,683,211 paired-end amplicon reads, complete agreement across the overlap identified 2,707,801 (73.52%) high quality sequences. Table S2 describes the number of reads that failed by sample, read direction, and the number of undetermined residues (Ns) present. For comparison, we analyzed the same 33 samples with quality filtering methods that rely on Q-scores to identify low quality reads described by Bokulich *et al.* [5] and Minoche *et al.* [6]. Quality filtering based upon Q-scores identified, on average, similar percentages (86–88%) of passed reads whereas the complete overlap analysis identified an average pass rate of 63% across all samples (Figure 2). Despite the great variation between the numbers of pairs in each individual sample (min: 6,785; max: 828,243, mean: 111,613), the ratio of reads that were identified as low quality was stable among the samples for each method (Figure 2).

**Figure 3:**
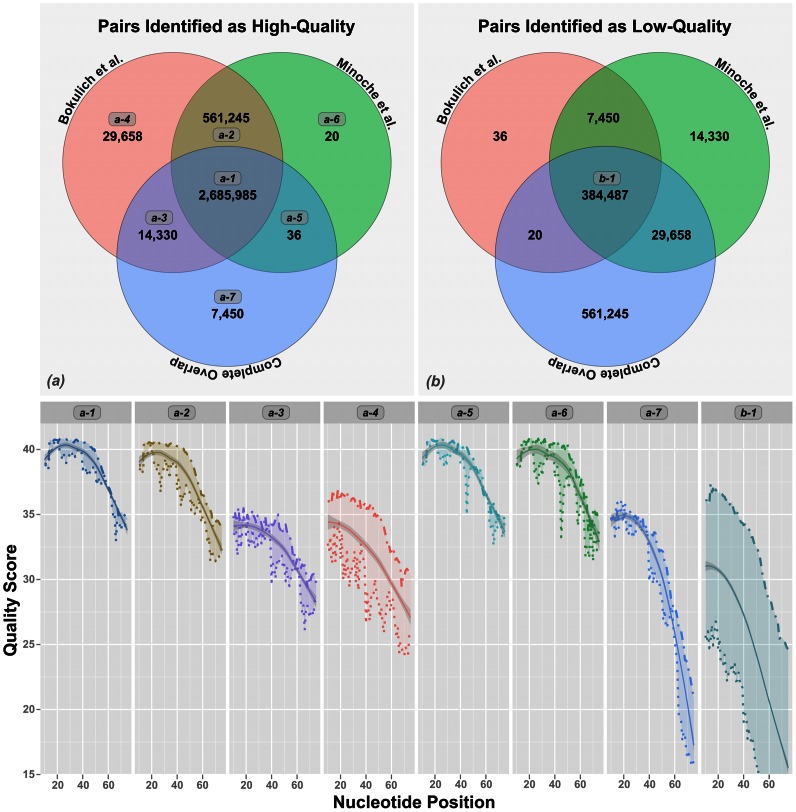
Paired-end reads from 33 samples that passed and failed the quality filtering by individual methods are compared in Venn diagrams. The mean quality scores of paired-end reads from the numbered regions in Venn diagrams are shown below. In each panel, the top and bottom lines show read 1 and read 2, respectively. The mean quality of each pair at each nucleotide position is also shown with a smooth line.

To explain the difference in numbers, we examined the fate of paired-end reads that the different filtering approaches identified as low or high quality. Our analysis showed that both Q-score-based analysis methods classified 561,245 paired-end reads as high-quality, that complete overlap analysis rejected as low-quality. Similarly, Q-score based methods rejected 7,450 paired-end reads that complete overlap identified as having high quality (Figure 3). With its default parameters, Bokulich identified 29,658 additional high-quality pairs that Minoche and complete overlap analysis rejected (Figure 3A). On the other hand, Minoche rejected 14,330 pairs that both Bokulich and complete overlap analysis identified as high-quality (Figure 3B).

**Figure 4:**
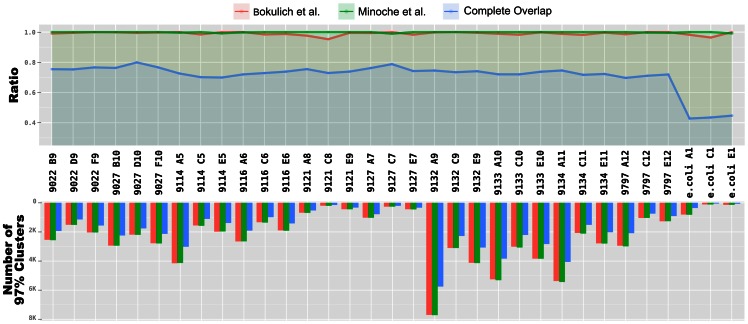
Comparison of the relative cluster counts and the absolute number of clusters identified in reads filtered by three different methods. The top panel shows the relative number of 97% OTUs for each method, with the method that produced the largest number assigned a value of 1.0. The bottom panel presents the actual number of OTUs for each method.

We clustered each sample at 97% to compare the number of OTUs in each sample for each of the three filtering methods (97% similarity is equivalent to 1 nt difference between ∼62 nt V6 reads). The number of OTUs identified in Bokulich and Minoche filtered reads yielded a remarkably similar number of OTUs for each sample, and the ratio of the number of clusters equaled to an average of 0.99 (standard deviation (*SD*) = 0.09) for Bokulich over Minoche. In contrast, the number of OTUs identified in reads filtered by the complete overlap method were consistently smaller with an average ratio of 0.70 (*SD* = 0.03) (Figure 4). The relative number of OTUs did not change at neighboring clustering thresholds of 96% (2 nt difference) (Bokulich/Minoche ratio of 0.99 (*SD* = 0.03); complete overlap ratio of 0.71 (*SD* = 0.12)) and 100% (0 nt difference) (Bokulich/Minoche ratio of 0.99 (*SD* = 0.02)); complete overlap ratio of 0.68 (*SD* = 0.03). Raw numbers of clusters for each sample are reported in Table S3.

## Discussion

Requiring perfect compliance between paired reads generates high-quality short sequences from Illumina paired-end reads from libraries that contain small amplicons or short insert genomic libraries. Short reads generated by this method contain a remarkably small number of random sequencing errors. In order for a read containing a random sequencing error to pass the filtering, not only must the location of the error be the same, but also the nucleotide changes must be complementary, which together render the probability very low. However, this method is incapable of reducing the number of PCR errors that occur during the original library amplification or the amplification during the cluster formation on the Illumina flow cell.

Our analyses with the V6 region of the 16S rRNA gene generated ∼62 nt sequences from each high quality paired-end read. ∼62 nt is equivalent to ∼30.7% of the information in a pair of two 101 nt reads. Following quality filtering, the final dataset only contained 22.56% of the information generated by the sequencer. The great reduction in the throughput makes overlapping region-based filtering methods unsuitable for projects where the main goal is the maximum throughput and/or maximum read length. However, they are promising for studies that demand very high accuracy.

Q-score-based quality filtering methods rely on PHRED-like algorithms [11], [12] to determine the accuracy of base calls. Even though the arbitrary Q-score thresholds recommended by empirical studies seem to be relatively successful at removing very low quality reads (Figure 3), methods that rely on Q-scores can over-estimate the accuracy. Overall, the number of clusters identified in reads filtered with complete overlap method was approximately 30% less than the number of clusters identified in reads that were filtered by Q-scores-based methods for each sample (Figure 4).

Comparison of the complete overlap analysis with two recently published quality filtering approaches by Bokulich *et al.* [5] and Minoche *et al.* [6] showed that while they perform well at identifying most low-quality reads, they tend to identify as high-quality many reads that contain random sequencing errors revealed by the complete overlap analysis (Figure 3). This is not because they are implemented poorly, but because Q-scores can be misleading. For instance, area *a-1* in Figure 3 shows the number of reads that were identified as high quality by all three methods. In contrast, area *a-*2 shows reads that were identified as high quality only by the two Q-score-based methods. The similarity between these two areas with respect to the mean quality scores (Figure 3, bottom panel) shows the difficulty of identifying reads that contain random sequencing errors via Q-score-based approaches alone. This emphasizes the importance of relying on methods that incorporate knowledge of the experimental design, e.g. degree of overlap or the existence of technical replicates, when the read quality is of utmost priority. The contribution of false positives (reads wrongly identified as high-quality) becomes clear when resulting reads are used for cluster analysis. After subsampling to the smallest number of reads, clustering analysis consistently resulted in more OTUs for reads quality-filtered by score based approaches.

The Bokulich method [6] performs very much like Minoche [5], especially with the suggested default parameters (*p* = 0.75, *q* = 3, *n* = 0, *r* = 3). With these parameters, Bokulich trims any read from where the first 3 consecutive bases (*r* = 3) remain below Q3 (*q* = 3), then, if the resulting read is not less than 75% of the original read in length (*p* = 0.75), and has 0 ambiguous bases (*n* = 0), it identifies it as a high quality read. Minoche *et al.* performs a similar step by removing B-tails (group of low quality, ambiguous bases at the end of reads). When *n* = 0 for Bokulich, which is the default behavior for Minoche, both methods remove any read containing an ambiguous base after quality trimming. The major difference between two approaches is the extra step of “Q33” filtering performed by Minoche. This algorithm calculates the length of the “B-tail” trimmed reads and retains the read only if two thirds of bases in the first half of the read have Q-scores over Q30. The sequences removed by “Q33” filtering explains most of the discrepancy between two approaches with respect to the number of reads that are identified as low quality. In our analyses, Bokulich identified 29,658 paired-end reads as high quality (area *a-4* in Figure 3), which were recognized as low quality by both Minoche and complete overlap analysis. Figure 3 shows the noticeable difference between mean Q-scores between area *a-4* and area *a-1*. However, the clustering analysis results indicate that despite the more sophisticated utilization of Q-scores by Minoche, the number of OTUs observed with either of these methods is very similar, and they perform similarly well at reducing noise. Although the Minoche trimming algorithm was optimized for trimming shotgun genomic sequences, it proves to be suitable for amplicon sequence quality filtering.

None of the quality filtering methods used in this study are able to reduce PCR error, which is an outstanding issue [13]. However, the ability to remove the majority of random sequencing errors improves the accuracy of bacterial diversity estimates. Although we used V6 region to demonstrate library preparation and quality filtering, this complete overlap approach can also be used to generate very high quality reads from shotgun metagenomic libraries. With the increasing read lengths of Illumina platforms (e.g., 250 nt paired-end reads on the MiSeq), it can be used to obtain longer high quality reads across multiple hypervariable regions of the 16S rRNA gene.

## Supporting Information

Table S1 Combination of 967F-AQ, 967F-UC3, 967F-PP and 967F-UC12 primers.
(DOC)

Table S2 Number of reads that failed during the filtering process for each method by sample, read direction, and the number of undetermined residues (Ns) present.
(XLS)

Table S3 Number of clusters identified at 96%, 97% and 100% sequence similarity thresholds in reads filtered by Bokulich, Minoche and Complete Overlap methods.
(XLSX)

## References

[1] Pedros-Alio C (2007) Ecology. Dipping into the rare biosphere. Science 315: 192–193. 17218512 10.1126/science.1135933

[2] Sogin ML, Morrison HG, Huber JA, Mark Welch D, Huse SM, et al (2006) Microbial diversity in the deep sea and the underexplored "rare biosphere". Proc Natl Acad Sci U S A 103: 12115–12120. 16880384 10.1073/pnas.0605127103 PMC1524930

[3] Huse SM, Welch DM, Morrison HG, Sogin ML (2010) Ironing out the wrinkles in the rare biosphere through improved OTU clustering. Environ Microbiol 12: 1889–1898. 20236171 10.1111/j.1462-2920.2010.02193.x PMC2909393

[4] Kunin V, Engelbrektson A, Ochman H, Hugenholtz P (2010) Wrinkles in the rare biosphere: pyrosequencing errors can lead to artificial inflation of diversity estimates. Environ Microbiol 12: 118–123. 19725865 10.1111/j.1462-2920.2009.02051.x

[5] Bokulich NA, Subramanian S, Faith JJ, Gevers D, Gordon JI, et al (2013) Quality-filtering vastly improves diversity estimates from Illumina amplicon sequencing. Nat Methods 10: 57–59. 23202435 10.1038/nmeth.2276 PMC3531572

[6] Minoche AE, Dohm JC, Himmelbauer H (2011) Evaluation of genomic high-throughput sequencing data generated on Illumina HiSeq and genome analyzer systems. Genome Biol 12: R112. 22067484 10.1186/gb-2011-12-11-r112 PMC3334598

[7] Zhou HW, Li DF, Tam NF, Jiang XT, Zhang H, et al (2011) BIPES, a cost-effective high-throughput method for assessing microbial diversity. ISME J 5: 741–749. 20962877 10.1038/ismej.2010.160 PMC3105743

[8] Masella AP, Bartram AK, Truszkowski JM, Brown DG, Neufeld JD (2012) PANDAseq: paired-end assembler for illumina sequences. BMC Bioinformatics 13: 31. 22333067 10.1186/1471-2105-13-31 PMC3471323

[9] Caporaso JG, Bittinger K, Bushman FD, DeSantis TZ, Andersen GL, et al (2010) PyNAST: a flexible tool for aligning sequences to a template alignment. Bioinformatics 26: 266–267. 19914921 10.1093/bioinformatics/btp636 PMC2804299

[10] Edgar RC (2010) Search and clustering orders of magnitude faster than BLAST. Bioinformatics 26: 2460–2461. 20709691 10.1093/bioinformatics/btq461

[11] Ewing B, Green P (1998) Base-calling of automated sequencer traces using PHRED. II. Error probabilities. Genome Res 8: 186–194. 9521922

[12] Ewing B, Hillier L, Wendl MC, Green P (1998) Base-calling of automated sequencer traces using PHRED. I. Accuracy assessment. Genome Res 8: 175–185. 9521921 10.1101/gr.8.3.175

[13] Lee CK, Herbold CW, Polson SW, Wommack KE, Williamson SJ, et al (2012) Groundtruthing next-gen sequencing for microbial ecology-biases and errors in community structure estimates from PCR amplicon pyrosequencing. PLoS One 7: e44224. 22970184 10.1371/journal.pone.0044224 PMC3435322

