# Correction: A Filtering Method to Generate High Quality Short Reads Using Illumina Paired-End Technology

**DOI:** 10.1371/annotation/afa5c40d-c604-46ae-84c4-82cb92193a5e

**Published:** 2013-06-25

**Authors:** A. Murat Eren, Joseph H. Vineis, Hilary G. Morrison, Mitchell L. Sogin

Cycling conditions were: an initial 94^o^C, 3 minute denaturation step; 30 cycles of 94^o^C for 30s, 60^o^C for 45s, and 72^o^C for 60s; and a final 2 minute extension at 72^o^C.

