# Supplementary material for: A Filtering Method to Generate High Quality Short Reads Using Illumina Paired-End Technology
Source: PLoS One. 2013 Jun 17;8(6):e66643. doi: 10.1371/journal.pone.0066643 (PMC3684618; doi:10.1371/journal.pone.0066643)
Supplement: Table S1 — Combination of 967F-AQ, 967F-UC3, 967F-PP and 967F-UC12 primers. (DOC) [file pone.0066643.s001.doc]

| Primer Name | | Primer sequence (5' to 3') |
| --- | --- | --- |
| 967F_PP_N4TCAGC | AATGATACGGCGACCACCGAGATCTACACTCTTTCCCTACACGACGCTCTTCCGATCTNNNNTCAGCCNACGCGAAGAACCTTANC | |
| 967F_AQ_N4TCAGC | AATGATACGGCGACCACCGAGATCTACACTCTTTCCCTACACGACGCTCTTCCGATCTNNNNTCAGCCTAACCGANGAACCTYACC | |
| 967F_U2_N4TCAGC | AATGATACGGCGACCACCGAGATCTACACTCTTTCCCTACACGACGCTCTTCCGATCTNNNNTCAGCCAACGCGMARAACCTTACC | |
| 967F_U3_N4TCAGC | AATGATACGGCGACCACCGAGATCTACACTCTTTCCCTACACGACGCTCTTCCGATCTNNNNTCAGCATACGCGARGAACCTTACC | |
| 967F_PP_N4GTATC | AATGATACGGCGACCACCGAGATCTACACTCTTTCCCTACACGACGCTCTTCCGATCTNNNNGTATCCNACGCGAAGAACCTTANC | |
| 967F_AQ_N4GTATC | AATGATACGGCGACCACCGAGATCTACACTCTTTCCCTACACGACGCTCTTCCGATCTNNNNGTATCCTAACCGANGAACCTYACC | |
| 967F_U2_N4GTATC | AATGATACGGCGACCACCGAGATCTACACTCTTTCCCTACACGACGCTCTTCCGATCTNNNNGTATCCAACGCGMARAACCTTACC | |
| 967F_U3_N4GTATC | AATGATACGGCGACCACCGAGATCTACACTCTTTCCCTACACGACGCTCTTCCGATCTNNNNGTATCATACGCGARGAACCTTACC | |
| 967F_PP_N4GCTAC | AATGATACGGCGACCACCGAGATCTACACTCTTTCCCTACACGACGCTCTTCCGATCTNNNNGCTACCNACGCGAAGAACCTTANC | |
| 967F_AQ_N4GCTAC | AATGATACGGCGACCACCGAGATCTACACTCTTTCCCTACACGACGCTCTTCCGATCTNNNNGCTACCTAACCGANGAACCTYACC | |
| 967F_U2_N4GCTAC | AATGATACGGCGACCACCGAGATCTACACTCTTTCCCTACACGACGCTCTTCCGATCTNNNNGCTACCAACGCGMARAACCTTACC | |
| 967F_U3_N4GCTAC | AATGATACGGCGACCACCGAGATCTACACTCTTTCCCTACACGACGCTCTTCCGATCTNNNNGCTACATACGCGARGAACCTTACC | |
| 967F_PP_N4ACGCA | AATGATACGGCGACCACCGAGATCTACACTCTTTCCCTACACGACGCTCTTCCGATCTNNNNACGCACNACGCGAAGAACCTTANC | |
| 967F_AQ_N4ACGCA | AATGATACGGCGACCACCGAGATCTACACTCTTTCCCTACACGACGCTCTTCCGATCTNNNNACGCACTAACCGANGAACCTYACC | |
| 967F_U2_N4ACGCA | AATGATACGGCGACCACCGAGATCTACACTCTTTCCCTACACGACGCTCTTCCGATCTNNNNACGCACAACGCGMARAACCTTACC | |
| 967F_U3_N4ACGCA | AATGATACGGCGACCACCGAGATCTACACTCTTTCCCTACACGACGCTCTTCCGATCTNNNNACGCAATACGCGARGAACCTTACC | |
| 967F_PP_N4GAGAC | AATGATACGGCGACCACCGAGATCTACACTCTTTCCCTACACGACGCTCTTCCGATCTNNNNGAGACCNACGCGAAGAACCTTANC | |
| 967F_AQ_N4GAGAC | AATGATACGGCGACCACCGAGATCTACACTCTTTCCCTACACGACGCTCTTCCGATCTNNNNGAGACCTAACCGANGAACCTYACC | |
| 967F_U2_N4GAGAC | AATGATACGGCGACCACCGAGATCTACACTCTTTCCCTACACGACGCTCTTCCGATCTNNNNGAGACCAACGCGMARAACCTTACC | |
| 967F_U3_N4GAGAC | AATGATACGGCGACCACCGAGATCTACACTCTTTCCCTACACGACGCTCTTCCGATCTNNNNGAGACATACGCGARGAACCTTACC | |
| 967F_PP_N4GACTC | AATGATACGGCGACCACCGAGATCTACACTCTTTCCCTACACGACGCTCTTCCGATCTNNNNGACTCCNACGCGAAGAACCTTANC | |
| 967F_AQ_N4GACTC | AATGATACGGCGACCACCGAGATCTACACTCTTTCCCTACACGACGCTCTTCCGATCTNNNNGACTCCTAACCGANGAACCTYACC | |
| 967F_U2_N4GACTC | AATGATACGGCGACCACCGAGATCTACACTCTTTCCCTACACGACGCTCTTCCGATCTNNNNGACTCCAACGCGMARAACCTTACC | |
| 967F_U3_N4GACTC | AATGATACGGCGACCACCGAGATCTACACTCTTTCCCTACACGACGCTCTTCCGATCTNNNNGACTCATACGCGARGAACCTTACC | |
| 967F_PP_N4CTAGC | AATGATACGGCGACCACCGAGATCTACACTCTTTCCCTACACGACGCTCTTCCGATCTNNNNCTAGCCNACGCGAAGAACCTTANC | |
| 967F_AQ_N4CTAGC | AATGATACGGCGACCACCGAGATCTACACTCTTTCCCTACACGACGCTCTTCCGATCTNNNNCTAGCCTAACCGANGAACCTYACC | |
| 967F_U2_N4CTAGC | AATGATACGGCGACCACCGAGATCTACACTCTTTCCCTACACGACGCTCTTCCGATCTNNNNCTAGCCAACGCGMARAACCTTACC | |
| 967F_U3_N4CTAGC | AATGATACGGCGACCACCGAGATCTACACTCTTTCCCTACACGACGCTCTTCCGATCTNNNNCTAGCATACGCGARGAACCTTACC | |
| 967F_PP_N4CGCTC | AATGATACGGCGACCACCGAGATCTACACTCTTTCCCTACACGACGCTCTTCCGATCTNNNNCGCTCCNACGCGAAGAACCTTANC | |
| 967F_AQ_N4CGCTC | AATGATACGGCGACCACCGAGATCTACACTCTTTCCCTACACGACGCTCTTCCGATCTNNNNCGCTCCTAACCGANGAACCTYACC | |
| 967F_U2_N4CGCTC | AATGATACGGCGACCACCGAGATCTACACTCTTTCCCTACACGACGCTCTTCCGATCTNNNNCGCTCCAACGCGMARAACCTTACC | |
| 967F_U3_N4CGCTC | AATGATACGGCGACCACCGAGATCTACACTCTTTCCCTACACGACGCTCTTCCGATCTNNNNCGCTCATACGCGARGAACCTTACC | |
| 1046R_index_1 | CAAGCAGAAGACGGCATACGAGATCGTGATGTGACTGGAGTTCAGACGTGTGCTCTTCCGATCTCGACRRCCATGCANCACCT | |
| 1046R_index_2 | CAAGCAGAAGACGGCATACGAGATACATCGGTGACTGGAGTTCAGACGTGTGCTCTTCCGATCTCGACRRCCATGCANCACCT | |
| 1046R_index_3 | CAAGCAGAAGACGGCATACGAGATGCCTAAGTGACTGGAGTTCAGACGTGTGCTCTTCCGATCTCGACRRCCATGCANCACCT | |
| 1046R_index_4 | CAAGCAGAAGACGGCATACGAGATTGGTCAGTGACTGGAGTTCAGACGTGTGCTCTTCCGATCTCGACRRCCATGCANCACCT | |
| 1046R_index_5 | CAAGCAGAAGACGGCATACGAGATCACTGTGTGACTGGAGTTCAGACGTGTGCTCTTCCGATCTCGACRRCCATGCANCACCT | |
| 1046R_index_6 | CAAGCAGAAGACGGCATACGAGATATTGGCGTGACTGGAGTTCAGACGTGTGCTCTTCCGATCTCGACRRCCATGCANCACCT | |
| 1046R_index_7 | CAAGCAGAAGACGGCATACGAGATGATCTGGTGACTGGAGTTCAGACGTGTGCTCTTCCGATCTCGACRRCCATGCANCACCT | |
| 1046R_index_8 | CAAGCAGAAGACGGCATACGAGATTCAAGTGTGACTGGAGTTCAGACGTGTGCTCTTCCGATCTCGACRRCCATGCANCACCT | |
| 1046R_index_9 | CAAGCAGAAGACGGCATACGAGATCTGATCGTGACTGGAGTTCAGACGTGTGCTCTTCCGATCTCGACRRCCATGCANCACCT | |
| 1046R_index_10 | CAAGCAGAAGACGGCATACGAGATAAGCTAGTGACTGGAGTTCAGACGTGTGCTCTTCCGATCTCGACRRCCATGCANCACCT | |
| 1046R_index_11 | CAAGCAGAAGACGGCATACGAGATGTAGCCGTGACTGGAGTTCAGACGTGTGCTCTTCCGATCTCGACRRCCATGCANCACCT | |
| 1046R_index_12 | CAAGCAGAAGACGGCATACGAGATTACAAGGTGACTGGAGTTCAGACGTGTGCTCTTCCGATCTCGACRRCCATGCANCACCT | |
